# Impact of Pay for Performance on Prescribing of Long-Acting Reversible Contraception in Primary Care: An Interrupted Time Series Study

**DOI:** 10.1371/journal.pone.0092205

**Published:** 2014-04-02

**Authors:** Myat E. Arrowsmith, Azeem Majeed, John Tayu Lee, Sonia Saxena

**Affiliations:** Department of Primary Care and Public Health, School of Public Health, Imperial College London, United Kingdom; NHS lothian and University of Edinburgh, United Kingdom

## Abstract

**Background:**

The aim of this study was to evaluate the impact of Quality and Outcomes Framework (QOF), a major pay-for-performance programme in the United Kingdom, on prescribing of long-acting reversible contraceptives (LARC) in primary care.

**Methods:**

Negative binomial interrupted time series analysis using practice level prescribing data from April 2007 to March 2012. The main outcome measure was the prescribing rate of long-acting reversible contraceptives (LARC), including hormonal and non hormonal intrauterine devices and systems (IUDs and IUSs), injectable contraceptives and hormonal implants.

**Results:**

Prescribing rates of Long-Acting Reversible Contraception (LARC) were stable before the introduction of contraceptive targets to the QOF and increased afterwards by 4% annually (rate ratios  = 1.04, 95% CI = 1.03, 1.06). The increase in LARC prescribing was mainly driven by increases in injectables (increased by 6% annually), which was the most commonly prescribed LARC method. Of other types of LARC, the QOF indicator was associated with a step increase of 20% in implant prescribing (RR =  1.20, 95% CI =  1.09, 1.32). This change is equivalent to an additional 110 thousand women being prescribed with LARC had QOF points not been introduced.

**Conclusions:**

Pay for performance incentives for contraceptive counselling in primary care with women seeking contraceptive advice has increased uptake of LARC methods.

## Introduction

Long-acting reversible contraception (LARC) is highly clinically effective compared with more widely used short term contraceptive methods. LARC is defined as ‘methods that require administering less than once per cycle or month[1], including non-hormonal Intrauterine Devices (Copper-IUD); hormonal IUD or Intrauterine Systems (IUS), also known as Levonorgestrel intrauterine systems(LNG-IUS); injectable contraceptives and hormonal implants. LARC has lower failure rates of <1% with typical use than oral contraceptive pills or condoms (8% and 15% respectively), partly due to easier compliance [2], [3] and becomes more cost-effective after the first year [1], [4]–[6]. Although LARC methods such as copper-intrauterine devices (copper-IUDs) are the most commonly used non-permanent contraceptives in the world, the use of LARC including IUDs is lower in many developed countries including the United Kingdom (UK) and the United States (US), both of which have high unintended pregnancy rates[7]–[10].

Recently in the UK, several government initiatives have aimed to increase awareness and use of LARC including public policy initiatives such as National Institute for Health and Clinical Excellence (NICE) LARC guidelines and Quality and Outcome Framework (QOF)[1], [11]. The pay-for-performance programme, implemented in April 2004 by the National Health Services (NHS), linked GP income to performance against targets set in QOF indicators [12]. A new set of Quality and Outcome Framework (QOF) indicators on contraception was introduced in the 2009-10 General Medical Services (GMS) contract for General Practices. Unlike previous years' QOF indicators in sexual health (CON1 and CON2) where the focus was only on having a policy for emergency contraception requests and provision of pre-conceptual advice[13]. Sexual health indicators (SH1, SH2, SH3) introduced in April 2009 focused on provision of information on long acting reversible methods of contraception to women attending for contraceptive advice[11] aimed at increasing awareness of LARC methods among women seeking contraceptive advice in general.

Previous studies evaluating the impact of QOF on care quality and health outcomes across different chronic disease areas have reported mixed results and several studies suggest improvements predate the QOF [14]–[17]. There is little evidence assessing the impact of QOF implementation on contraceptive provision. This study investigates the impact of QOF contraceptive incentives on LARC uptake by examining LARC prescribing patterns in primary care before and after the introduction of QOF contraception indicators in 2009.

## Methods

### Data

Data used in this study were obtained from the Prescription Pricing Authority (PPA) which is the main source of information on general practitioners' prescribing in England. The specific data record we used is the Prescribing Analysis and Cost (PACT) data which records all NHS prescriptions issued by general practitioners and dispensed by pharmacists and has been widely used for research[18].

We obtained quarterly prescribing data from a random sample of 581 general practices in England from April 2007 to March 2012. Our sample can be seen as nationally representative, as we compared our sample practices with the national data for practice characteristics including the registered female population 15 to 44 years old, locality (urban/rural), deprivation (Index of Multiple Deprivation), number of GPs and number of female GP and ethnicity (percentage of White British registered population) and found no statistically significance between our sample and the population except for the ethnicity in which the national average was 80% compared with 71% in our sample.

To preserve confidentiality, the PPA was not able to supply data from single-handed practices. NHS prescribing outside of general practices including prescriptions from hospitals that were dispensed in the community, prescriptions dispensed in hospitals or mental health units and private prescriptions were not included.

We grouped contraceptive prescribing data as combined oral contraceptive pills (COCs), progestogen-only pills (POPs), injectables, Intra-uterine devices (IUDs) copper and progestogen-containing, implants, diaphragm and caps, spermicides, contraceptive patches, vaginal rings and emergency hormonal contraceptives (EHC). EHC were excluded from the analysis as our main outcome of interest was on-going use of contraceptives not emergency use. We defined LARC as IUDs, implants and injectables and non-LARC consists of COCs, POPs, diaphragms and caps.[19]

### Outcome measure

Our primary outcome measure was the rate of GP prescribed LARCs to their registered population. The basic measure of volume is the number of prescription items, where items refer to a single item on a prescription form. To enable comparison between LARC and non-LARC items, namely COCs, and POPs, we transformed the items into annual prescribed units for patients. We adjusted prescribing rates converting monthly prescriptions to equivalent items per annum for COCs and POP and 4 injectable items per annum (since these are administered every 3 months). LARC methods were assumed to be one item per patient. We calculated contraceptives prescribed per 1000 registered female population aged 15–44 years to account for variation between practices.

### Statistical analyses

We used a negative binomial interrupted time-series (ITS) method to estimate changes in levels and trends in prescribing of LARCs after of the QOF contraceptive services incentives were implemented. While taking into account the overall time trend, this model estimates both the immediate change and change in time trend after the policy implementation [15], [20]–[24]. To account for unobserved heterogeneity for general practice, we fitted our data using a random effect model. We calculated changes in prescribing rate ratio (RR) which is the ratio of the actual prescribing rate in relation to the rate projected by the underlying trend.

Our model was adjusted for underlying time trend (continuous variable for each quarter), seasonal effect (dummy variables for quarters in financial year), geographical region (dummy variable for PCTs), ethnicity, residence (urban/rural) and socioeconomic index for each practice. We used the Index of Multiple Deprivation (IMD), provided by the Department of Communities and Local Government as a deprivation measure, deriving categorical quintiles of deprivation for IMD where the highest quintile was set as the most deprived. We derived a measure of rurality for each practice location based on population density of the practice postcode calculated from the 2001 census by Office for National Statistics (ONS). We also included in our model the presence of at least one female GP in the practice as a dummy variable, since having a female GP selectively increases provision of methods such as IUDs [25], [26].

We estimated number of additional LARC prescriptions after the implementation of QOF by estimating the number of prescription in the absence of the policy (the counterfactual). To do this, we used the coefficient estimated in the model but setting the policy dummy and time after the policy variables to zero for the whole time period, then adding the differences in number of prescription each quarter between the actual prescription and counterfactual estimate.

We tested for multicollinearity for covariates controlled for in our analysis. The multicollinearity diagnostics (VIF) were all less than 5, indicating that multicollinearity was not a problem. We tested for linearity of the time trend and added a quadratic term for the time variable when the linearity of the time trend was not met. Additionally, we conducted a sensitivity analysis to assess whether any changes in prescribing were also found in non-LARCs items. We performed all statistical analyses using Stata 11 (StataCorp).

## Results

There was wide variation across practices in the numbers of contraceptives prescribed, numbers of registered women aged 15 to 44 years and QOF achievements. The mean number of registered patients per practice was 1577 (range 54 to 8868) and mean QOF contraception achievement in 2010-11 was 82% (range 0 to 100%) and 89% (range 0 to 100%) in 2011-12. The most commonly prescribed contraceptives were COCs at 38 per 1000 registered women aged 15 to 44 years (range 0 to 122), followed by injectables, POPs, implants and IUS, at 17 (range 0 to 182), 13 (range 0 to 51), 2 (range 0 to 44) and 2 (0 to 30), respectively. Copper-IUDs (IUCD) were the least commonly prescribed LARCs in the primary care at 1 per 1000 registered females aged 15–44 years (range 0 to 13).

### Prescribing rates before and after QOF

**Figure 1:**
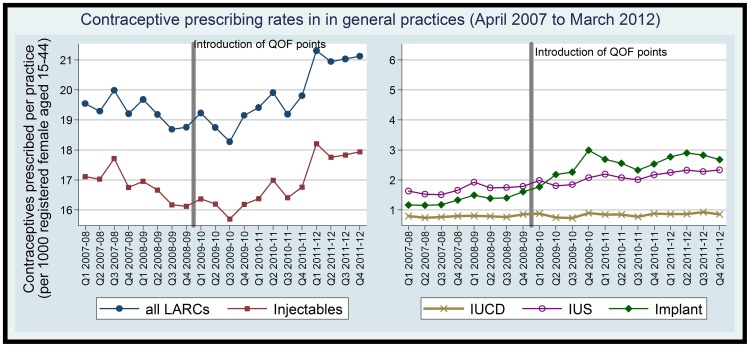
prescribing rate in general practices for registered women aged 15–44 years.

LARC prescribing was stable prior to April 2009, with a decreasing gradient of −0.4% annually (Table 1). This changed to an increasing trend in LARC prescribing of 4% annually (adjusted rate ratio = 1.04, 95% CI =  1.03, 1.06) after the introduction of QOF contraception incentives. Overall, the mean number of LARC prescribed by practices increased by 10% in the 4 years after-QOF contraception indicators were implemented compared to the pre-QOF baseline in 2008-09. Of the main LARC methods we found prescribing of implants increased substantially by 88% whilst IUS, injectable, IUCD and increased by 27%, 8%, and 8% respectively (Figure 1).

**Table 1:** Interrupted time-series analysis of LARC prescribing rates in general practices.

	LARC (95% CI)	Injectable (95% CI)	Implant (95% CI)	IUS (95% CI)	IUCD (95% CI)
**Time**	0.99 (0.98,1.02)	0.98 (0.96,0.99)	1.64 (1.49,1.80)	1.19 (1.11,1.28)	1.04 (0.98,1.11)
**QOF**	0.99 (0.97, 1.02)	0.99 (0.96, 1.01)	1.20 (1.09, 1.32)	0.97 (0.90, 1.04)	1.02 (0.94, 1.10)
**Time after QOF**	1.04 (1.03, 1.06)	1.06 (1.04, 1.08)	1.34 (1.13, 1.58)	1.11 (0.97, 1.26)	0.99 (0.93, 1.05)
**Seasonality**					
**Apr-Jun**	–	–	–	–	–
**Jul-Sep**	0.97 (0.96, 0.99)	0.98 (0.97, 0.99)	0.95 (0.91, 1.00)	0.93 (0.89, 0.96)	0.95 (0.90, 1.01)
**Oct-Dec**	0.97 (0.95, 0.98)	0.98 (0.96, 0.99)	0.93 (0.88, 0.98)	0.91 (0.88, 0.95)	0.94 (0.89, 1.00)
**Jan-Mar**	0.98 (0.96, 0.99)	0.98 (0.97, 1.00)	1.02 (0.97, 1.07)	0.94 (0.91, 0.98)	1.01 (0.95, 1.07)
**Socioeconomic status**					
**Quintile 1 (the most affluent)**	–	–	–	—	—
**Quintile 2**	1.21 (1.05, 1.39)	1.30 (1.11, 1.51)	1.32 (1.08, 1.60)	1.02 (0.81, 1.29)	1.21 (0.91, 1.61)
**Quintile 3**	1.33 (1.14, 1.55)	1.49 (1.26, 1.75)	1.23 (0.98, 1.54)	0.75 (0.57, 0.97)	1.40 (1.01, 1.93)
**Quintile 4**	1.64 (1.40, 1.93)	1.81 (1.52, 2.16)	1.14 (0.91, 1.44)	0.65 (0.49, 0.85)	1.10 (0.79, 1.54)
**Quintile 5 (the most deprived)**	1.05 (0.88, 1.25)	1.04 (0.86, 1.25)	1.26 (0.96, 1.65)	0.43 (0.32, 0.59)	1.07 (0.74, 1.56)
**Residence**					
**Rural**	—	—	—	—	—
**Urban**	0.77 (0.66, 0.91)	0.84 (0.71, 0.99)	0.43 (0.35, 0.54)	0.55 (0.42, 0.72)	0.45 (0.32, 0.63)
**White British (%)**	1.01 (1.00, 1.01)	1.01 (1.00, 1.01)	1.00 (1.00, 1.01)	1.01 (1.00, 1.01)	1.00 (0.99, 1.00)
**Female GP in the Practice**					
**Without Female GP**	–	–	–	–	–
**With Female GP**	2.03 (1.82, 2.27)	1.81 (1.61, 2.04)	2.40 (1.95, 2.97)	1.81 (1.46, 2.25)	2.26 (1.76, 2.90)

Note: LARCs (Long-Acting Reversible Contraceptives), IUS (Intra-uterine system), IUCD (Intra-uterine copper devise).

‡ Model also controlled for regional variables (Primary Care Trust dummy variables), results not shown.

‡ As the linearity of the time trend was not met for implant and IUS outcomes, quadratic term for the time variable was added in the model.The beta coefficients are 0.91 (95% CI = 0.89, 0.94) for implant, and 0.97 (95% CI = 0.95, 0.99) for IUS.

The increase in LARC prescribing was driven by increases in injectables, which were the most commonly prescribed LARC method. Injectables increased by 6% annually (RR =  1.06, 95% CI =  1.04, 1.08) in the post-QOF period against a decreasing secular trend −2.4% (RR =  0.98, 95% CI =  0.96, 0.99). There was a step change in prescribing rate of implants, which increased by 20% increase (RR =  1.20, 95% CI =  1.09, 1.32) immediately after the QOF indicators were introduced. The trend change (RR = 1.34, 95% CI = 1.13, 1.58) suggested that there was a sustained increase in implants prescribing in the post-QOF period when compared to the pre-QOF period.

The oral contraceptive prescribing rate was increasing by 1.4% (RR =  1.01, 95% CI 1.01, 1.02) before QOF and decreased by 1.2% annually (RR =  0.99, 95% CI =  0.98, 0.99) in the post-QOF implementation period. POPs prescribing rates were increasing by 12% (RR =  1.12, 95%CI =  1.11, 1.13) before QOF implementation then decreased by 3% annually afterwards(RR =  0.97, 95% CI =  0.97, 0.98). The COCs prescribing rate remained the same with a 2% decreasing trend before and after QOF.

### Practice characteristics associated with LARC prescribing

GPs in urban practices were less likely to prescribe LARC methods than rural GPs, by 23% (RR =  0.77, 95% CI =  0.66 to 0.91), and this was most significant for implants, IUS and IUCD methods, 57% (RR =  0.43, 95% CI =  0.35, 0.54), 45% (RR =  0.55, 95% CI =  0.42, 0.72) and 55% (RR =  0.45, 95% CI =  0.32, 0.64) respectively. The presence of one or more female GPs in a practice was associated with a doubling in LARC prescribing compared to those with no female GP in the practice (RR = 2.03, 95% CI = 1.82, 2.27). This was particularly significant for IUCD and implants (RR =  2.26, 95% CI = 1.76, 2.90; and 2.40, 95% CI = 1.95 to 2.97 respectively).

## Discussion

Prescribing rates of LARC methods increased by 4% annually after the QOF contraceptive indicators were implemented. This increase is equivalent to 8700 more women being prescribed with LARC (1100 in the first year, 3200 and 4400 in the second and third year) after the implementation of QOF contraception incentives. Extrapolating this effect to the nationally registered population of women aged 15 to 44 years this is equivalent to an additional 110 thousand women who would have been prescribed LARC. The increase was accompanied by falls in oral contraceptive pill prescribing indicating there might have been a switch to long term contraceptive methods during the post-QOF period.

### Strengths and weaknesses in relation to other studies

This study is the first to investigate the impact of QOF on contraceptive provision. The interrupted time series method that we used disentangled the secular time trend from the effect of the QOF. We found no published studies on contraceptive prescribing trends during the time frame of the observed LARC increases against which to validate our estimates. Previous studies evaluating the effectiveness of QOF on prescribing of other drugs have shown similar increases in the volume of prescribing after the introduction of the QOF in both England and Northern Ireland compared to the period before[27]. Some have suggested that QOF may have rewarded and reinforced existing prescribing behaviours [28]. Our findings are congruent with several previous studies on the impact of QOF showing an immediate impact of QOF, which then plateaued [14]–[16] [29]–[32]. However there are a number of important limitations relating to data quality and completeness. Routinely collected data from PACT is not subject to quality control for research purposes and we did not carry out any internal validation. As with any observational time trend analysis, the observed increases are related to specific time points surrounding the introduction of QOF contraception indicators. Although we adjusted to the Primary Care Trust level within our model, we did not have information regarding local initiatives to improve access to LARC. We also did not have information on the influence of pharmaceutical companies over the same time frame through marketing, promotion and provision of training on fitting of their LARC products [33], [34]. As with any ecological study design evaluating the changes of a national policy the changes observed in the time frame against the background trends may in part be explained by the effect of concurrent efforts to increase LARC for example as a result of social marketing.

During the study period, the commonest type of implant, Implanon, was replaced by Nexplanon. The decrease in supply and retraining required may have exerted a downward pressure on the overall trend during the course of this changeover. Another factor is that the Faculty for Reproductive and Sexual Health published more stringent guidance on the training requirements for GPs who inserted IUS/IUCD's. These tighter monitoring requirements may also have exerted a downward pressure on prescribing that could have diluted the effect of our results of the potential impact of the QOF itself.

Our study is also subject to ecological fallacy in that our observed increase in practice level LARC prescribing may not have increased specifically in those women given contraceptive advice and recorded as having met the QOF contraceptive targets. As our data from e-PACT excluded single-handed practices, we cannot extrapolate findings to this group. This potentially introduces a selection bias. However, the sensitivity analysis of characteristics of our final sample of women aged 15 to 44 years was nationally representative.

### Policy implications and future research recommendations

Recent research on contraceptive method choice among European women found that the decision to use IUDs over other methods is dictated by recommendations from the physician rather than women [35]. The majority of unintended pregnancies have been attributed to contraceptive failure and given that most women in the UK access contraception from their GPs [36], a switch to more clinically effective methods is likely to lead to a substantial decrease in unintended pregnancies [37]–[40].

There may have been a wide variation between practices on how QOF contraceptive care ‘quality’ indicators were achieved that will affect the effectiveness of policy in future. Misconceptions about side effects of many contraceptive methods including LARC are common amongst UK general practitioners [41], [42]. The quality of contraceptive discussions can range from simply handing over a leaflet to interactive in-depth discussion of each method with the patient. Contraceptive decision making itself is a complex process where women's existing ideas and concerns affect their acceptance of LARC [25], [39]. Not all practices will have a trained doctor or practice nurse who can administer or fit LARC methods. GPs who are trained to fit IUDs or implants are more likely to offer the method. There may have been an accompanying increase in provision of LARC methods in sexual reproductive health clinics in the UK after QOF indicators were introduced due to referrals by practices who do not provide LARC within the practice. As our data did not have information for LARC items issued by family planning and sexual health clinics, we might underestimate the true impact of the QOF.

It should be noted that the definition of LARC for the purposes of the QOF included injectable hormonal contraceptives. Injectable methods have a far lower continuation rate (50%) compared with implants and IUDs (75 to 80%) and are not effective in reducing unplanned pregnancy unlike other LARCs[3], [4]. Arguably these should not be offered as a LARC method and possibly should even be dropped from the QOF definition. Economic and resource implications for wider evaluation of this policy include cost-effectiveness of the additional consultation time required to achieve the QOF indicators, the training needs for LARC fitting in general practices and use of contraceptive services in the community.

These incentives targeted women already consulting for contraceptive services, whose compliance, and risk of unplanned pregnancy may differ from other groups of sexually active women who do not take regular contraception, or those presenting for termination counselling. In future, the target population could be widened to include all women of child bearing age, which might require call and recall systems rather than opportunistic counselling. Any such change would carry with it ethical and resource considerations that would need to be carefully evaluated. The discontinuation rate and the reason for discontinuation would be important outcomes in assessing the quality of discussions given that there may be improvement with continuations for those who received in-depth discussions [43]. We therefore recommend further research to investigate whether an increase in uptake of LARC in primary care leads to a reduction of unintended pregnancies in the long-term.

## Conclusion

Our findings suggest that pay for performance incentives for contraceptive counselling in primary care with women who are on oral contraceptives or those requesting emergency contraception have increased uptake of the more effective LARC methods. Widening this policy has potential to result in a reduction in unintended pregnancy but has resource, training and cost implications. However, more information is needed on its acceptability, sustainability, cost effectiveness and long term benefit on reproductive outcomes including continuation rates with LARC methods and impact on unplanned pregnancy.
